# The association between visual attention and body movement-controlled video games, balance and mobility in older adults

**DOI:** 10.1186/s12877-021-02358-9

**Published:** 2021-06-30

**Authors:** Mansour Alghamdi, Lori Ann Vallis, Susan Jennifer Leat

**Affiliations:** 1 Department of Optometry, College of Applied Medical Sciences, King Saud University, PO BOX 68953, Riyadh, Riyadh 11537 Saudi Arabia; 2 School of Optometry and Vision Science, University of Waterloo, 200, University Ave. West, Waterloo, ON N2L 3G1 Canada; 3 Department of Human Health & Nutritional Sciences, University of Guelph, Guelph, ON N1G 2W1 Canada

**Keywords:** Visual attention, Balance, Mobility, Video games, Gait, Useful field of view, Multiple object tracking, Body movement-controlled video games, Microsoft™ Xbox® 360 Kinect™

## Abstract

**Background:**

Body movement-controlled video games involving physical motion and visual attention may have the potential to train both abilities simultaneously. Our purpose was to determine the associations between performance in these games and visual attention, balance and mobility in a group of older adults. The long-term goal is to identify the optimal type of interactive games with regards to training potential.

**Methods:**

Fifty healthy adults aged 65+ years participated in this cross-sectional study. Visual attention was measured with static and dynamic versions of a useful field of view (UFV) and a multiple object tracking (MOT) test. Balance was measured with a force plate in bi-pedal quiet stance test (QST) and one-legged stance (OLST). Gait variability and walking speed were assessed with the Five Meter Walk Test (5MWT). Four Microsoft™ Xbox® 360 Kinect™ interactive video games were chosen based on the apparent levels of visual attention demand.

**Results:**

Visual attention (UFV and MOT) was significantly associated with performance in Xbox® Kinect™ games that appeared to have a high visual attention demand (*p* < 0.05), while there was minimal or no significant association with games with apparent low visual attention demand. Balance and mobility show correlations with visual attention, and with Xbox games.

**Conclusion:**

The results suggest that there are relationships between visual attention, balance, mobility and Xbox® Kinect™ game performance. Since different Xbox® games were associated with different balance, mobility and visual attention scores, a variety of such games, rather than a single game, may be most effective for training for falls prevention.

## Introduction

Experiencing a fall can pose a serious threat to the safety and health of an older adult. Falls are the leading injury resulting in death worldwide [60]. In Canada there is an increase in prevalence among seniors who are 65 years or older [45, 53] which is representative of the global falls epidemic. Falls are also the leading cause of partial or total permanent disability due to injury in this older age group [49, 53]. Thirty percent of adults > 65 years fall once per year [26] and this rises to 50% in those who are over 80 years of age [30]. Among older adults who fall, 20 to 30% have moderate to severe injuries that lead to serious health impacts or even to death [2, 52]. The impact of a falls is not confined only to the injury itself because, even with no reported physical injury there can be a loss of confidence that leads to a decrease of activities which may lead to future falls [2, 13, 17].

Falls are multifactorial [2, 54, 62]. The most pertinent to the current study are the internal biological factors related to falls, specifically vision and balance control. The control of balance is an integrative process between three sensory systems: visual, vestibular and somatosensory [37]. Some studies indicate that visual input is the most salient, particularly for mobility [24, 35]. The role of visual input in maintaining balance and preventing a fall has been extensively studied [7, 37]. In particular, visual acuity, visual field, contrast sensitivity, glare sensitivity, and depth perception often have been studied and shown to be main aspects of vision that are associated with falls [7, 24, 37, 38]. Recent studies have included another important aspect of vision, visual attention, and have shown a relationship with balance [1] and mobility [34, 41]. These studies used useful field of view (UFV) tests to measure attention, which are well-researched [4, 51] and improve with training [51]. The UFV can document delays in the processing time of the sensory information [5, 55, 56] or errors during divided attention tasks [10, 33]. These delays and errors may result in negative effects on the postural control system, i.e. loss of balance and/or a fall.

Video gaming has markedly increased in popularity, driven in part with new developments in visual technology [20, 23]. Recently, research has examined the potential of this technology for falls prevention and falls rehabilitation purposes [19, 42, 43, 47, 48]. Traditionally, exercise is an important component of falls prevention and rehabilitation programmes [18] and recent research findings have shown that Nintendo Wii balance board (WBB) or Xbox® Kinect™ can be used to present a unique and fun environment to assess and train the physical performance of both young and older people [19, 42, 43, 47, 48]. For example, performance on Nintendo Wii balance board (WBB) correlates with balance measures and exercising with such games can improve balance [19, 42]. These video games create a virtual reality scene on a computer or television screen and the player then alters their body position to interact with the virtual environment. In the Nintendo WBB games, the player’s movements are detected by a balance board controller (the WBB) which enables them to control the game avatar’s movements. Unfortunately, the size of balance board may present a challenge to some players due to the small base of support provided and may also limit a player’s movement as the player cannot step beyond the board.

These limitations are not applicable with systems that use a body motion sensor (called body movement-controlled video games**)**, such as Xbox® 360® Kinect™, which can track body posture and motion in free space [6, 57]. The advantage of using kinematic based motion sensors is that the player can move and exercise over a more extensive space, making it possible to include a wider range of physical activities. Body movement games, such as Xbox® Kinect™, may have an additional benefit over straightforward exercise, providing simultaneous physical exercise and vision attention training as they provide simultaneous physical exercise and vision attention training, which in turn provides synergic benefits on cognitive and brain functioning [12] and may help to reduce fragility in the elderly [9].

The relationship between video games and visual attention has been addressed in some research studies. Video game players show better performance in selective attention and divided attention as measured with UFV [20, 22] and are also able to successfully track a greater number of objects for a MOT task [15, 20, 21]. The degree of visual attention training would likely be optimised with games that include a high visual demand.

The purpose of this study is to determine the potential of this type of training to improve visual attention, balance and mobility in a group of older adults and based on these findings, choose the optimal type of Xbox® games to facilitate the largest gains in these areas for future studies. Xbox® Kinect™ games were chosen that appeared to have a stronger or a weaker visual attention requirement. We hypothesize that performance on the games that appear to have high visual attention demand will be predicted by tests of visual attention in comparison to games with low apparent visual demand. We are also interested to study the associations between visual attention and measures of mobility and balance. Therefore, the present study also aimed to determine the relationships between visual attention, balance, mobility and performance in Xbox® Kinect™ games.

## Methods

The study was reviewed and received clearance through a University of Waterloo Research Ethics Committee (ORE 20689) and was conducted according to the Declaration of Helsinki guidelines.

### Subjects

This cross sectional study took place at the University of Waterloo School of Optometry and Vision Science between January 2015 and June 2017. Fifty participants aged 65+ years were recruited from the University of Waterloo Optometry Clinic and from among staff and faculty, and their friends and family members at the School of Optometry and Vision Science, University of Waterloo. We also used “snowball” recruiting where participants were asked if they knew of friends or family who might be eligible and willing to participant.

Inclusion criteria for all participants were: aged 65 and above, either biological sex, relatively good health (see below), Montreal Cognitive Assessment (MoCA) test score after correction for level of education ≥24 [40, 50], not using medications which are a known the risk for falls (see below), independently mobile (able to walk without a cane or walking frame), no clinical vision loss (described below), and no previous use of Xbox® exercise gaming. Participants with a diagnosis of the following were excluded: dementia, Parkinson’s disease, history of cerebrovascular accident resulting in residual paresis, multiple sclerosis, cerebellar dysfunction, peripheral neuropathy of any etiology, advanced arthritis so as to cause significantly reduced range of motion of the weight bearing or small joints, or significant hearing loss. Use of medications is expected to be high in this age group, so we only excluded participants who used medications which may increase the risk of falls or impair balance (i.e., antipsychotics, sedatives, antidepressants, anti-histamines, anti-hypertensive, and long-acting sleeping medications). For vision, all participants had binocular visual acuity 6/12 (20/40) or better, with no diagnosed glaucoma or hemianopia.

Our sample size was similar to that reported in previous similar correlation studies. Bowers (2013; sample size *N* = 32) found r = 0.36 and 0.50 between UFV and MOT, Leat and Lovie-Kitchin [34] sample size *N* = 35) found r between 0.3 and 0.62 between AFV and various aspects of mobility, Althomali and Leat ([1] sample size *N* = 72) found r = 0.4 between balance (one-legged stance test) and UFV Reed-Jones et al. (2011; sample size *N* = 34) found correlations of 0.24 to 0.34 between UFV measures and Wii balance. We also performed a sample size calculation. Based on these studies set our statistical significance acceptance to be a value of r = 0.45, alpha = 0.05, power = 80%, which required a total of 36 study participants for our experimental paradigm. We increased this to fifty.

### Procedures

#### Screening for inclusion criteria

A questionnaire included questions about general and ocular health, and medications, which was administered either by phone or in person. The Montreal Cognitive assessment (MoCA) (www.mocatest.org) was administered in the usual way, with the exception that the letter T was used instead of F for the Language component. The result was corrected for the level of education and the exclusion criterion was chosen as < 24 [40].

Visual acuity was measured binocularly with the participant’s habitual spectacles, defined as those that the participant used for driving, walking and shopping. Visual acuity (VA) was measured with the ETDRS visual acuity chart “R”, available from Precision Vision (www.precision-vision.com), at 4 m [16]. The chart luminance was between 80 and 120 cd/m^2^. Visual acuity was measured in logMAR using by-letter scoring [3, 27].

Monocular visual field screening was conducted for each eye in order to confirm that there was no large field defect of which the participant was unaware. A confrontation test was used, the participant being asked to count fingers presented in each field quadrant [14].

#### Balance measures

Participants were asked to undertake two different sets of balance assessments: bi-pedal quiet stance (QST) and the one-legged stance test (OLST). Both balance tests were performed with the eyes open, fixating on a target in front of them; each balance assessment condition was performed three times. The QST required them to stand quietly (without moving or talking) on a portable force plate (200 Hz; AccuGAIT, AMTI, Inc) with the feet placed approximately shoulder width apart for one minute. The portable force plate measures ground reaction forces and moments under the feet and facilitates the calculation of the centre of pressure (CoP; the weighted average of the pressure underneath the feet; [44]. Participants were required to lightly clasp their hands together in front for the duration of the trial. For OLST the participant was asked to stand still on his/her preferred leg with the other leg extended in front for 30 s with their hands held at their sides.

To reduce the impact of initial and final acclimatization periods on the force plate, only the middle 50 s for the quiet stance trials and 20 s for single legged stance trials were used to calculate the centre of pressure (i.e. the first and final 5 s were deleted for each trial). For each participant, the maximum anterior-posterior (AP) and medial-lateral (ML) Co*P* values and range were calculated. The path length in centimeters (cm) was then calculated for each time point using Pythagorean theorem from CoP anterior-posterior (AP) and medial-lateral (ML) sway values. From this data, the cumulative path length (CPL) was calculated (sum of the resulting path length vector, over time). The standard deviation of each of these measures for each person was calculated to give a measure of their postural variability. Variability is a measure of balance control; in general, high variability in CoP measures for an individual is indicative of poor overall balance control and an increased risk for falls [39].

#### Mobility/gait test

A Five-Meter Walking Test (5MWT) was used to assess walking speed and gait variability for all participants. Participants wore their comfortable walking shoes and preceded to walk back and forth (~ 13-m pathway) on a hard floor, covered with a strip of paper which was taped to the floor, for a total of 2 walking trials. Before they walked, stickers were attached to the posterior heel of their shoes [58] that were subsequently covered with ink, to mark their steps. The length of the paper was 9 m and the width was 65 cm. Two meters (about three steps) was added at the beginning and at the end of the paper walkway to facilitate the examination of gait parameters during the steady state stage of gait only, i.e. the acceleration phase (gait initiation) and the deceleration phase (gait termination) were not included in the analyses. All participants were instructed to walk at a pace that they would normally use when shopping.

The 5MWT measures were determined from the central 5 m for both directions of walking (approximately 7–8 steps in each direction). The average time for walking in both directions was calculated. Following completion of the two walking trials, step length and width (cm) was measured for each step from the ink marks from heel to heel in the direction of travel and perpendicular to the direction of travel, respectively [58]. The average and standard deviation of step length and width were then calculated for each step and averaged across the two walking trials. Lastly, an average of the walking velocity for both 5-m walks was calculated (5 m/time to complete) and then normalized to the leg length via the calculation of a ratio (velocity/leg length, VL). This ratio adjusted participants’ gait velocity for their leg length (measured from greater trochanter of the femur to the floor) to facilitate comparisons across different participants.

#### Visual attention tests

Spatial selective visual attention was measured using a useful field of view (UFV) test [4, 32, 34, 51, 59] and spatial, sustained visual attention was measured using a Multiple Object Tracking test [8]. All the visual attention tests were presented on 23.6-in LED monitor at a viewing distance of 50 cm. To focus on this working distance, participants were given + 1.75D over-the-counter reading glasses to wear (over their habitual distance glasses if they had them).

**Fig. 1:**
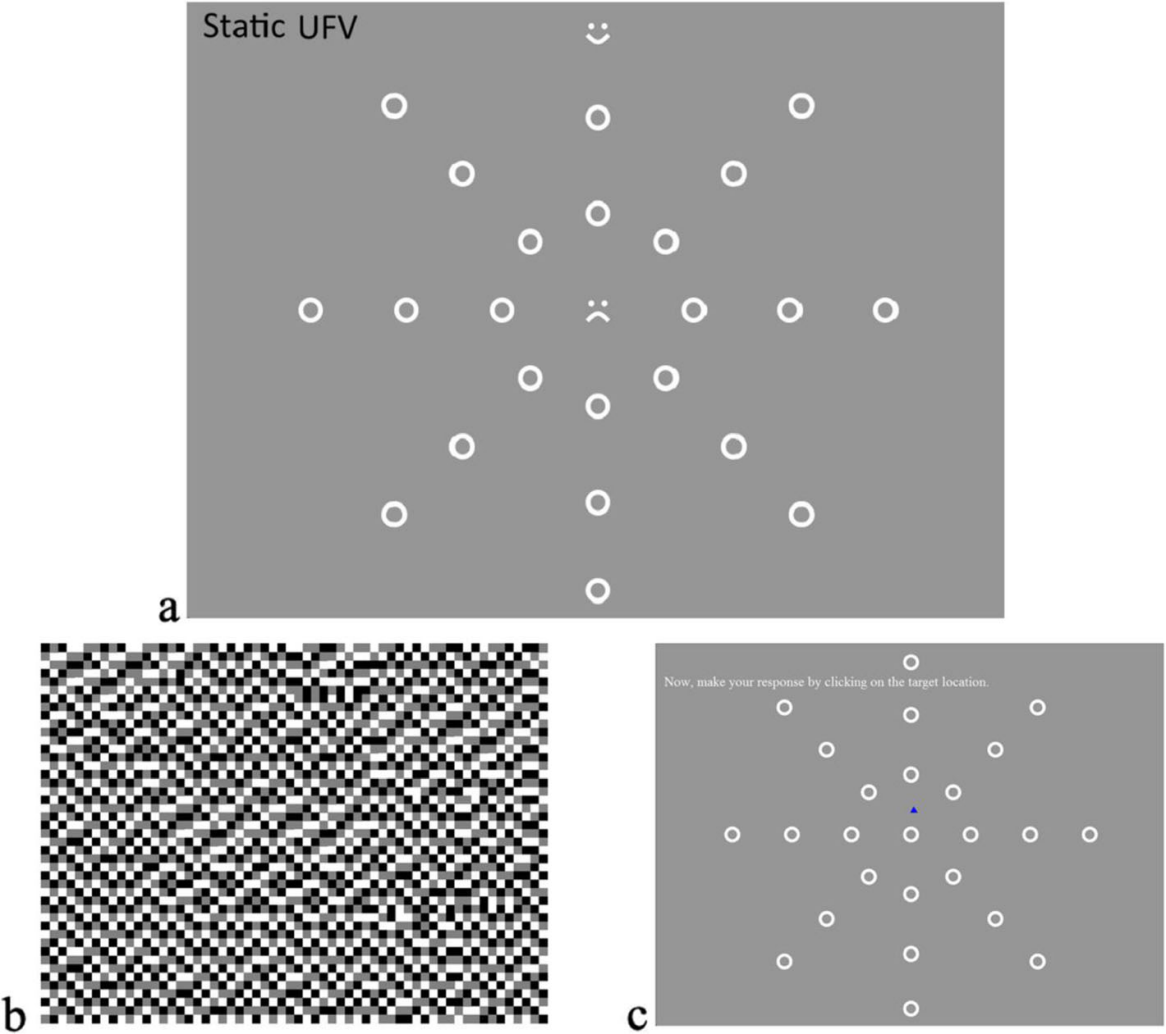
The static useful field of view (UFV-S) a) stimulus in which a central target (smiling or frowning face) and peripheral target (smiling face) are simultaneously presented among distractors (circles) for 200 ms. b) mask presented after the stimulus to eliminate any after-image. c) response screen where participant had to verbally identify the central target first then point to the location of the peripheral target

There were two versions of the useful field of view tests: static and dynamic. The static version (UFV-S) was similar to condition 4 (selective attention) in Leat et al. [34] but with different targets [59]. The central task was to identify if the target was either a smiling or frowning face. The peripheral target was a ‘smiley face’ located among circular distractors (Fig. 1)(a). The size of the whole display subtended 30^°^ by 30^°^ at the 50 cm viewing distance. There were 24 distractors arranged in three concentric circles (10^°^, 20^°^, 30^°^) along eight axes. The diameter of each distractor and target was 1.26^°^ (11 mm) in diameter and the line width was 0.23^°^ (2 mm). The peripheral target was presented twice in each location and the order of all presentations was randomised, therefore, there was a total of 48 trials, with each trial presentation lasting 200 ms. After each trial, a mask screen was shown to avoid any after-image effects (Fig. 1)(b). Then the participant had to verbally identify the central target and point to the location of the peripheral target on the response screen. The trial was considered correct if the participant was able to correctly identify the central target and accurately locate the peripheral target. Participants received audible feedback for each correct response. The outcome measure of this test was accuracy, in percent.

**Fig. 2:**
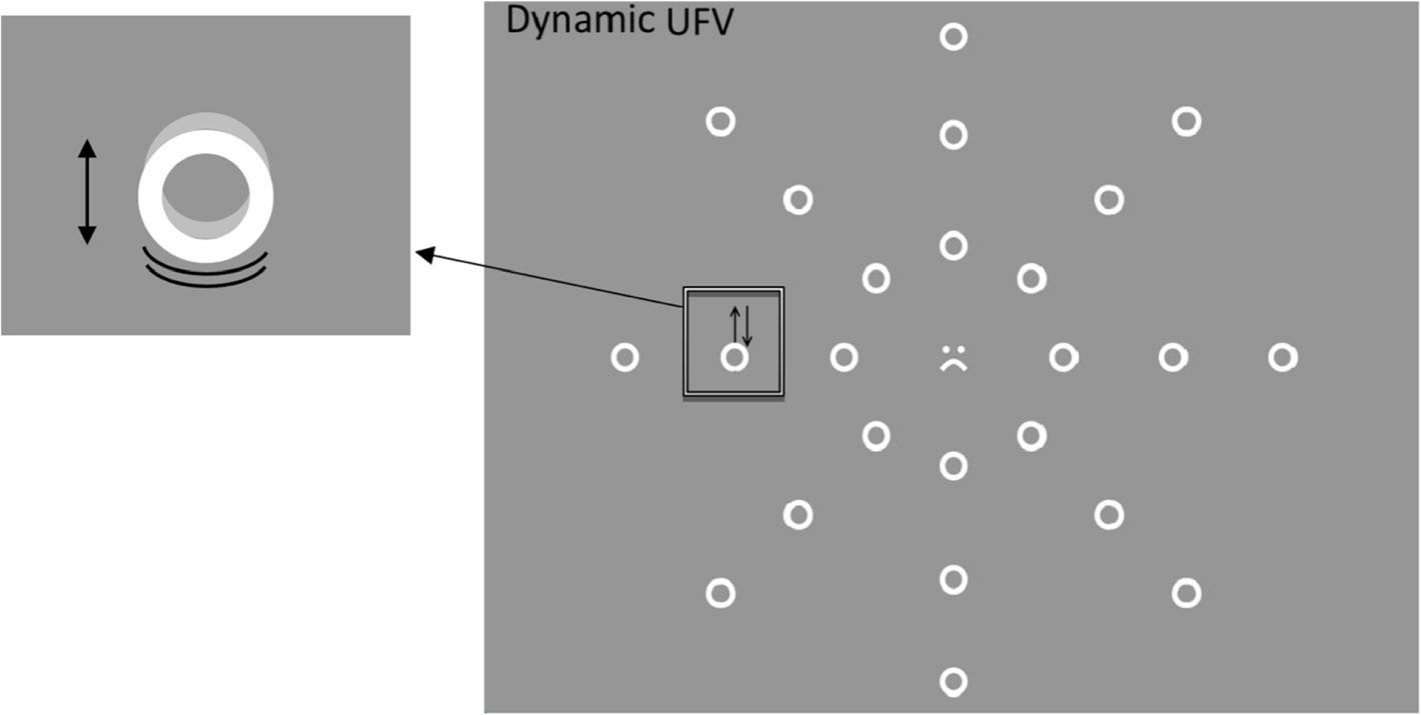
The dynamic useful field of view (UFV-D), showing the stimulus screen with the central target (smiling or frowning face). The target is one of the peripheral circles which moves up and down during the presentation time (200 ms). The inset illustrates the movement. The light grey circle represents the maximum extent of movement away from its initial position, shown by the white circle

The dynamic version of the UFV was developed as it was thought that detecting movement might be more associated with ability to detect moving objects or targets in the periphery during the Xbox® games. This test used the same procedure as the UFV-S but differed in the peripheral target. Instead of a smiley face, one of distractor circles moved upwards by 0.23^°^ and then returned to its position (one cycle up and down) during the presentation of each trial (Fig. 2). All other measures and outcomes were the same as the static UFV.

Before conducting the visual attention tests, each participant was given practice trials on both versions of UFV tests. To reduce the practice effect, there were no more than 10 practice trials for either static or dynamic UFV and the target duration was longer (500 ms) to give the participant more time to understand the test (without giving them practice at the actual test duration).

Sustained visual attention was measured with multiple object tracking (MOT) [46]. To reduce test duration, the brief MOT test was used [8]. The field size subtended 20^°^ (18.2 by 18.2 cm) at the 50 cm viewing distance. The stimuli were six black circles 1.5^°^ (1.31 cm) in diameter which moved randomly on a white background. There were three practice trials and 40 experimental trials. At the beginning of each trial three of the stimuli circles turned to yellow for 2 s and then turned to black again. These three yellow circles were considered as targets. The participant was required to track the three target circles for 5–8 s, at which point the circles stopped moving and the participant was asked to identify the targets. The trial was considered correct only if the participant was able to identify all three targets correctly. The first trial speed was always 12^°^ per second. The speed threshold was determined with a one up, one down staircase. The speed increased by 40% after a correct response and decreased 60% after an incorrect response. The outcome measure was the angular target speed to give a 60% correct threshold [8].

#### The Xbox® Kinect™ video games

The Xbox® 360® console with the Kinect™ controller was used for the video games. The Kinect sensor can recognise and localise the physical position and motion of the player. An avatar or virtual augmented image is created by the game and is controlled by the motion of the player. For all games, participants stood in front of a 39-in TV (89 by 52 cm) at a distance of two meters. The screen subtended 24^0^ horizontally at 2 m, which is where the participant started for each game. Four different games were chosen based on the apparent visual requirements. Two games appeared to have high visual demand (action games) and two appeared to have low visual demand (exercise games), chosen from Xbox®360 Kinect™ commercially available games called “Your shape™” and “Sports season2”.

The two apparently low visual demand games were Leg exercise and Zen energy (Tai Chi). In these games, participants followed an on-screen coach. In the Leg exercise, there were three exercise movements: step squat, sumo squat and side to side lunge. The movements aim to train the thigh muscles. In Zen energy, there were three main movements: side travel, ballet movement and warrior posture. These movements are meant to stretch the thigh muscles and enhance balance control. For both games, the movements were demonstrated by the experimenter and then the participant practiced them once before they following the on-screen coach. The Kinect sensor tracked the player’s movement and assessed their ability to copy the correct position. A score is given based on the accuracy with which they follow the coach’s movements, and the outcome measure was the final percentage score given in the software.

The two high visual demand games selected for study had more visual complexity and faster motion, which required faster reactions and movement in order to attain higher game scores. The games chosen were *Skiing* and *Stomp-it*. In these games, participants saw a digital avatar which mimics their movements. Participants were asked to control the avatar’s movement with their own body movements and to achieve the best possible score.

In *Skiing* the participant stood in front of the screen and mimicked downhill skiing movements, for example, they were asked to avoid virtual flags/gates and make jumps. As such, movements produced during the game by players require more coordinated movements of the upper and lower body; to obtain a high score quick reactions to the upcoming obstacles were required. After an explanation, the participant completed one practice trial using the game software and if there were no questions or concerns they then completed two different downhill runs. The outcome measure was the accuracy of performance in terms of successfully avoiding the flags and gates and making the jumps. Note that the participant did not have to actually jump (leave the ground) to make the avatar jump – they could just flex their knees to make a “sham” jump, and they were informed of this.

In *Stomp-it* colored panels start moving from right, left, front-right or front-left of the screen and move towards the avatar. The participant was required to step with one foot on each colored panel when it reaches the avatar. The number of correct steps during the trial is used to quantify the performance score of the participant. The participant was given an explanation and a practice trial before commencing the actual experimental trial.

The TV screen showing the avatar was videotaped for all games and this video recording was used afterward for final scoring. For *Skiing* and *Stomp-It* a scoring system was devised to that each error (e.g., gate hit or incorrect step) was counted equally. For the exercise games, the video was used, as the score for the game remained on the screen too briefly to record in real time.

The order of the visual attention tests and Xbox® games was balanced as follows. The participant always started with one visual attention test (UFV or MOT) followed by one set of Xbox® games (Tai Chi/Legs or Skiing/Stomp-It). Then the second visual attention test was followed by the second set of Xbox games set. The order of the specific attention tests and Xbox® games was counter-balanced between the participants using a block design and the order of the Xbox® games and UFV tests was alternated between participants (e.g., Tai Chi/Legs or Legs/Tai Chi, Static/Dynamic or Dynamic/Static UFV).

One person (MA) collected all data including the clinical measures (e.g. MoCa, one-legged stance test, visual acuity) which may have resulted in some bias. However, this minimized issues related to inter-rater reliability (e.g. instructions to participants) for these assessments. Additionally, balance measures were derived from forceplate data and calculated separated from gait metrics, which were computed and compiled in blocks subsequent to the participant’s visit.

### Data analyses

For data analyses, the UFV scores were arcsine transformed as is usual [34]. The data were tested for normalcy with the Kolmogorov-Smirnov test and Shapiro-Wilk test. Since a number of the measures were found not to be normally distributed, all the data were transformed by an arcsine transform (for those that were a percent correct) or a log transformation. After transformation, all the data was found to be normally distributed, except for number of medications or general health conditions. So, these variables (number of medications and general health conditions) were split into a two-way score. For medications this was 0 for no medications and 1 for one or more, and for general health this was zero for up to one condition and 1 for two or more co-morbidities. For ease of understanding, the results are reported as the raw results (untransformed data). The data were plotted as histograms and there were clear outliers for some measures. Outliers that were more than 3 standard deviations from the mean were excluded [29]. Note that there were no missing data in this data set.

### Statistical analyses

The data were first analysed with unadjusted univariate linear regression analyses to describe the proposed relationships between the variables of interest. There were three groups of correlations conducted; correlations within the visual attention tests, correlations of visual attention tests with video games and correlations of visual attention tests with balance and mobility outcome measures. Adjusted Bonferroni correction was applied to correct for multiple comparisons within each sub-analysis [31]. The univariate analysis was followed by linear regression adjusting for age and then age, gender, general health condition score and medications score.

Separate forward step-wise multiple regression analyses were conducted for video games and balance and mobility measures as dependent variables. A *p*-value of 0.05 to enter and 0.10 to remove was used. Since there was a high correlation among measures of attention, mobility and two-legged stance balance, one independent variable was selected from each of these groups of variables to enter the model. The one chosen from each group was that which had the highest correlation with the dependent variable. Many participants were not able to stand on one leg for thirty seconds for three trials. Thus, the outcome measure from this test used in our statistical model was the total one-legged stance time (OLST) for the three trials, resulting in only one measure for OLST. For each analysis, age, gender, general health, number of medications, MoCA and OLST were also included. For example, in the model to predict performance in Xbox® Skiing the following independent variables were entered; UFV-S (best visual attention measure), Velocity/Leg (best mobility measure), CoP ML-Max. (best balance measure), OLST, VA, gender, age, number of medications, general health and MoCA. Since we were interested in the association between performance in the tests and modifiable factors (which might be trained), in cases where non-modifiable factors, such as age or gender were predictors, the analysis was repeated without including these non-modifiable factors. A variance inflation factor (VIF) was calculated to ensure that the multiple regression models were not affected by multicollinearity. Data were analyzed with SPSS version 24 (Chicago, IL, USA) and a *p* value of < 0.05 was used for significance.

## Results

Fifty community–dwelling adults completed this study, 22 males and 28 females with an average age of 72.4 years ±5.1. Demographic data of the participants and average results for the tests of attention, gait, mobility and balance are shown in Table 1.

**Table 1:** Characteristic of Study Sample (*N* = 50)

Characteristic	Mean Value (SD)	Range
**Age** (years)	72.4 (5.1)	65–87
Male	73.1 (5.3)	65–87
Female	71.9 (5.1)	65–87
MoCA score	27.8 (1.5)	24–30
Number of medications	0.62 (0.9)	0–4
Number of co-morbidities	0.48 (0.7)	0–3
Visual Acuity in logMAR (VA)	−0.00 (0.06)	(−0.14) - 0.12
MOT (threshold speed, deg./sec.)	12.1 (4.1)	5.2–21.8
UFV-S (accuracy %)	36.8 (21.1)	2.1–83.3
UFV-D (accuracy %)	59.7 (24.4)	4.2–95.8
**Low visual demand games**		
Leg exercise (% correct)	52.4 (14.1)	5–78
Tai Chi (% correct)	41.4 (19.8)	8.2–79.2
**High visual demand games**		
Skiing (% accuracy)	70.8 (10.9)	42.2–93.8
Stomp-it (%)	36.2 (19.3)	0–94.1
**Mobility, mean ± SD**		
Step length (cm)	64 (8.2)	39.2–86.2
Step length variability (cm)	3 (1.1)	1.4–6.3
Step width (cm)	9.1 (3.4)	1.8–23
Step width variability (cm)	3.2 (1)	1.9–6.1
Stride length (cm)	129.7 (16.1)	80.5–171.7
Stride length variability (Right) (cm)	4.5 (2)	1.3–11.4
Five-meter walking time (secs)	4.6 (1)	3.2–8.3
Velocity/leg height	1.2 (0.2)	0.7–1.6
**Balance (cm), mean ± SD**		
ML COP SD	0.24 (0.1)	0.09–0.59
AP COP SD	0.37 (0.1)	0.21–0.74
ML COP MAX	0.59 (0.31)	0.19–1.68
AP COP MAX	0.96 (0.3)	0.51–2.28
ML COP Range	1.2 (0.6)	0.39–3.56
AP COP Range	1.94 (0.69)	1.04–5.41
Cumulative path-length	213.3 (80.3)	109.8–572.3
One-legged stance test (OLST) (secs)	73.2 (23.6)	0–90

*MoCA* the Montreal Cognitive Assessment, *MOT* the Multiple Object Tracking, *UFV-S* the Useful Field of View test- Static, *UFV-D* the Useful Field of View test – Dynamic, *COP* Centre of Pressure, *ML* Medial-lateral, *AP* Anterior-posterior, *MAX* Maximum

### Correlations with age

Age was correlated with all visual attention tasks (*p* < 0.05) except UFV-D and these remained significant after adjusted Bonferroni. For the games, one extreme outlier (> 3 SD from mean) was removed from the Stomp-It data before analysis. There was a significant association with age for Stomp-it (r = 0.4, *p* = 0.004), Tai Chi (r = − 0.36, *p* = 0.011) and Leg exercise (r = 0.28, *p* = 0.048) but no significant correlation of age with Skiing. These correlations remained significant after adjusted Bonferroni. Among mobility measures, walking speed, vel/leg and step length variability showed a significant correlation with age (r ≥ 0.31, p = ≤0.029) but these did not remain significant after adjusted Bonferroni. Finally, among balance measures, all the ML sway variables were significantly correlated with age (r ≥ 0.37, p = ≤0.0009), as was cumulative path-length (r = 0.35, *p* = 0.013). OLST was strongly correlated with age (r = 0.53, *p* < 0.001), All the balance measures remained significant after adjusted Bonferroni.

### Univariate analyses

The results of the unadjusted and adjusted univariate analyses of associations of the visual attention measures with mobility, balance and Xbox® games are shown in Table 2. Note that the significant correlations are included in this Table and those that are bolded are those correlations which remained significant after adjustment for age or age, number of medications and general health. Unadjusted univariate linear regression in the higher visual demand games (Skiing and Stomp-It) showed significant correlations with visual attention tests (MOT and UFV-S) (*p* = 0.003 and *p* = 0.026, respectively) although the correlation with Stomp-It was borderline after adjusted Bonferroni correction. None of the lower visual attention games was correlated with visual attention after adjusted Bonferroni. The correlation between Skiing and UFV-S remained after adjustment for age, medications and general health. For the mobility measures, the unadjusted univariate linear regression showed a significant association between step width variability and MOT and UFV-S (*p* = 0.004 and *p* = 0.044 respectively) which remained significant after adjusted Bonferroni. The association with MOT remained after adjustment for age, medications and general health. In term of balance results, one-legged total stance time (OLST) and ML CoP showed significant correlations with UFV-D (*p* = 0.007) and these remained significant after adjusted Bonferroni, but did not remain significant after adjustment for age.

**Table 2:** Unadjusted and adjusted Pearson correlation coefficients for visual attention against video games, balance and mobility. Only those that gave significant unadjusted correlations at the *p* = 0.05 level are included and those that remained significant after adjustment for age, and then age, no. of medications and general health are bolded. The astrix (*) indicates findings that remain significant after applying the adjusted Bonferroni correction [31]. Note that those showing negative correlation coefficients were expected as for one of the variables, a lower number means better performance

	Unadjusted r value (p)	Adjusted for age r value (p)	Adjusted for age, no. of medications and general health r value (p)
Xbox 360® Stomp-It with MOT	0.316 (0.026)	0.249 (0.074)	0.200 (0.179)
Xbox 360® Skiing with UFV-S	0.408 (0.003)*	**0.370** (0.011)*	**0.383** (0.015)*
Xbox 360® Tai Chi with UFV-S	0.294 (0.038)	0.198 (0.166)	0.184 (0.235)
Step Width (SD) with MOT	−0.402 (0.004)*	**− 0.377** (0.009)*	**− 0.367** (0.017)*
Step Width (SD) with UFV-S	− 0.285 (0.044)*	− 0.248 (0.098)	− 0.213 (0.185)
OLST with UFV-D	0.375 (0.007)*	**0.256** (0.042)	0.179 (0.175)
ML CoP (SD) with UFV-D	−0.301 (0.033)*	**−0.256** (0.042)	− 0.113 (0.447)

*MOT* Multiple Object Tracking, *OLST* One-Legged Stance Test, *UFV-S* Useful Field of View test- Static, *UFV-D* Useful Field of View test – Dynamic, *ML CoP* Medial-Lateral Centre of Pressure, *SD* Standard Deviation

Considering the associations between Xbox® games, and mobility and balance measures, only Tai Chi showed a significant correlation with balance (cumulative path length *p* = 0.009 and OLST *p* = 0.023) and mobility (mean stride length *p* = 0.03; 5MWT *p* = 0.001; Vel./Leg *p* < 0.001). These all remained significant after the adjusted Bonferroni. The association between Tai Chi and mobility remained after correction for age and age, GH score and medications. The other games did not show a significant association with any balance or mobility measures.

### Multiple regression models

Table 3 shows the multiple linear regression models for each of the Xbox® games together with the independent variables that were entered into the analysis. For Skiing accuracy, the only predictor was UFV-S. The Skiing accuracy increased by 0.264% for each 1 % increase in UFV-S accuracy. The model indicates that about 17% of the variability of Skiing accuracy can be explained by UFV-S. For Stomp-it, the only predictor was age, and performance in Stomp-it decreased 0.016% for each year of age. This model indicates that about 15% of the variability of performance in the Stomp-it can be explained by age. When the regression analysis was repeated excluding the non-modifiable factors of age and gender, MOT was the predictor. Performance in Tai Chi was only predicted by velocity corrected for leg length (Vel/Leg). About 24% of the variability in Tai Chi performance can explained by the Vel/Leg. The performance in Tai Chi increased 1.24% for each unit in Velocity/leg high ratio. Finally, performance in Leg exercise was predicted by age and performance in Leg exercise decreased 0.01% for each additional year of age. There were no predictors other than age which were significantly associated with leg exercise.

**Table 3:** Forward stepwise multiple linear regression between Xbox® games, (dependent variable) and visual attention and other variables. The variables that were entered into each model are shown beneath. The model for Stomp-It was run first with the full range of variables (full model), and secondly excluding the non-modifiable factors of age and gender

Dependent Variable	Predictor variable	R^**2**^ at each step	Co-efficient B	Standardized Coefficient	t	P value
Xbox360® Skiing^1^	UFV-S	0.167	0.264	0.408	3.1	0.003
*R*^*2*^ *for the model = 0.17, F = 9.61, p for the model = 0.003* ^1^predictors entered into the analysis: UFV-S, Vel/Leg, CoP ML Max, OLST, VA, gender, age, no. medications, general health and MoCA						
Xbox360® Stomp-It (full model)^2^	Age	0.146	−0.018	−0.381	−2.86	0.006
*R*^*2*^ *for the model = 0.15, F = 8.175, p for the model = 0.006* ^2^predictors entered into the analysis: MOT, step width average, Cumulative path-length, OLST, VA, gender, age, no. medications, general health and MoCA						
Xbox360® Stomp-It (excluding age and gender)^3^	MOT	0.1	0.48	0.316	2.3	0.026
*R*^*2*^ *for the model = 0.1, F = 5.309, p for the model = 0.026* ^3^predictors entered into the analysis: MOT, step width average, Cumulative path-length, OLST, VA, no. medications, general health and MoCA						
Xbox360® Tai Chi^4^	Vel/Leg	0.238	1.240	0.487	3.87	< 0.001
*R*^*2*^ *for the model = 0.24, F = 14.96, p for the model < 0.001* ^4^predictors entered into the analysis: UFV-S, Vel/Leg, Cumulative path-length, OLST, VA, gender, age, no. medications, general health and MoCA						
Xbox360® Leg exercises^5^	Age	0.079	−0.009	−0.281	−2.03	0.048
*R*^*2*^ *for the model = 0.08, F = 4.12 p for the model = 0.048* ^5^predictors entered into the analysis: UFV-S, 5MWT, CoP AP SD, OLST, VA, gender, age, no. medications, general health and MoCA						

*MoCA* the Montreal Cognitive Assessment, *MOT* the Multiple Object Tracking, *UFV-S* the Useful Field of View test- Static, *VA* Visual Acuity, *CoP* Centre of Pressure, *ML* Medial-lateral, *AP* Anterior-posterior, *MAX* Maximum, *5MWT* Five-Meter Walking Test, *OLST* One-Legged Stance Test, *Vel/Leg* Velocity/Leg length, *SD* Standard Deviation

Table 4 shows the step-wise multiple linear regressions for balance and mobility, respectively. The cumulative path-length was chosen as a good overall representation of bipedal stance. Performance in the one-legged stance test was predicted by age (*p* < 0.001), increase cumulative path-length (*p* = 0.003) and step length variability (*p* = 0.040), and when age was removed, cumulative path-length and step length variability remained as predictors. Poor balance as shown by the bipedal cumulative path-length was predicted by decreases in OLST (*p* = 0.004) and decreases of Velocity/leg height ratio (*p* = 0.025).

**Table 4:** Forward stepwise multiple linear regression for balance measures with visual attention and other variables. The variables that were entered into each model are shown beneath. The model for OLST was run first with the full range of variables (full model), and secondly excluding the non-modifiable factors of age and gender

Dependent Variable	Predictor variable	R^**2**^ at each step	Co-efficient B	Standardized Coefficient	t	P value
OLST (full model)^1^	Age Cumulative path-length Step length variability	0.283 0.408 0.460	− 0.032 − 0.979 − 0.78	−0.347 − 0.299 − 0.253	−3.727 −3.144 − 2.144	0.005 0.018 0.040
^1^predictors entered into the analysis: UFV-D, stride length variability, Cumulative path-length, VA, gender, age, no. medications, general health, and MoCA *R*^*2*^ *for the model = 0.46, F = 13.01 p for the model < 0.001*						
OLST (excluding age and gender) ^2^	Cumulative path-length Step length variability	0.267 0.359	−1.284 −1.015	−0.392 − 0.328	−3.107 − 2.597	0.000 0.013
^2^predictors entered into the analysis: UFV-D, stride length variability, Cumulative path-length, VA, no. medications, general health, and MoCA *R*^*2*^ *for the model = 0.36, F = 13.17 p for the model < 0.001*						
Cumulative pathlength^3^	OLST Vel/leg	0.267 0.343	−0.12 − 0.50	−0.517 − 0.3	−4.183 − 2.322	0.004 0.025
^3^predictors entered into the analysis: UFV-D, Vel/Leg, OLST, VA, gender, age, no. medications, general health and MoCA *R*^*2*^ *for the model = 0.343, F = 12.25 p for the model < 0.001*						

*MoCA* the Montreal Cognitive Assessment, *UFV-D* the Useful Field of View test- Dynamic, *VA* Visual Acuity, *OLST* One-Legged Stance Test, *Vel/Leg* Velocity/Leg length

For mobility (Table 5), velocity/leg ratio and the overall speed of walking the Five Meters Walking Test were chosen as good overall representations of mobility. Velocity/leg height ratio and the Five Meters Walking Test were both predicted by cumulative path-length.

**Table 5:** Forward stepwise multiple linear regression for mobility measures

Dependent Variable	Predictor variable	R^**2**^ at each step	Co-efficient B	Standardized Coefficient	t	P value
Velocity/leg height^1^	Cumulative path-length	0.211	−0.277	−0.459	−3.580	0.001
^1^predictors entered into the analysis: UFV-S, Cumulative path-length, OLST, VA, gender, age, no. medications, general health and MoCA *R*^*2*^ *for the model = 0.21, F = 12.82 p for the model = 0.001*						
Five Meters Walking Test^2^	Cumulative path-length	0.153	0.232	0.391	2.947	0.005
^2^predictors entered into the analysis: UFV-S, Cumulative path-length, OLST, VA, gender, age, no. medications, general health and MoCA *R*^*2*^ *for the model = 0.15, F = 8.686 p for the model = 0.005*						

*MoCA* the Montreal Cognitive Assessment, *UFV-S* the Useful Field of View test- Static, *VA* Visual Acuity, *COP* Centre of Pressure, *ML* Medial-lateral, *AP* Anterior-posterior, *MAX* Maximum

The regression models were not affected by multicollinearity as the variance inflation factor analyses (VIF) was less than 2.00 for all regression models in this study [25].

## Discussion

The main finding in this study is the expected correlation found between visual attention measures and high visual demand Xbox® games, especially Skiing. The correlation between Skiing and UFV-S remained even after adjusting for age, number of medications and general health status. The multiple-regression model for Skiing illustrates the importance of UFV-S in predicting the game performance as it was the only predictor in that model. However, the correlation co-efficient is low and only 17% of the variance was accounted for, which indicates that there are likely many factors which determine performance in the game.

Performance in Stomp-it was best predicted by age in the multiple regression model. However, when non-modifiable factors were removed, Stomp-It was predicted by visual attention (MOT). This is also shown in the simple correlation between Stomp-it and MOT, although this was borderline after adjusted Bonferroni and became non-significant after adjusting for age and age/health/medications. This indicates that both MOT and Stomp-It are determined by age and health. Although Skiing and Stomp-it have a high visual attention component, it is likely that Stomp-it requires more physical agility, as the participant has to step from one foot to the other quickly in response to the incoming colored targets. In Skiing, although the relative weight on each foot has to be changed, the participant does not have actually make a step. In other words, the greater physical demand in Stomp-it may overshadow the link with visual attention in the initial multiple-regression. Age itself is well correlated with the physical measures, such as balance and walking.

As predicted, our results demonstrate that games with apparent low visual demand such as Leg exercise or Tai Chi, show no correlation with visual attention tasks. The regression model of the low visual demand Xbox® games shows that these games are predicted only with either age or physical factors.

These findings are interesting because they illustrate that there may be potential of using these types of games for training visual attention and mobility/balance concurrently. As visual attention can be improved with training [4, 51], potential associations of visual attention with video games is important as video games might be used as a tool to train visual attention, with the expectation that this would transfer to other everyday life tasks including physical ability and cognitive status, which are also known to be associated with visual attention. However, to the authors’ knowledge there are no studies showing a correlation of body movement control games with visual attention tasks. Some studies have shown that playing sedentary action video games can improve aspects of functional vision such as crowded visual acuity [22], contrast sensitivity [36] visual field sensitivity [11], and visual attention tasks [15, 20, 21].

It has also been suggested that body movement video games such as Wii fit games or Xbox® Kinect™ games can be used useful in training physical abilities in older and younger people [19, 42, 43, 47, 48]. Our results show that the Tai Chi game was well correlated with physical abilities such as balance and mobility and that many balance/mobility measures are intercorrelated. This was consistent with another study which reported that Tai Chi training is correlated with balance and mobility [61]. Leg exercise, however, was not strongly correlated with either physical abilities or visual attention. Although these exercises games seem similar, the Tai Chi game is possibly more demanding in terms of the amount of movement/stretch required and the time to hold the pose.

Visual attention has been shown to have an association with mobility [1, 34, 41] and balance [1]. Our results are consistent with these previous results and show some correlation with gait and balance. Associations were observed in the current study between step width variability and MOT and UFV-S. The association with MOT remained even after adjustment for age, medications and general health status. Balance as measured by the OLST and the medial-lateral center of pressure variability was also associated with UFV-D. These correlations remain significant when adjusted for age but not when adjusted for age, number of medication and general health status. However, for balance and gait, in the multiple regressions, it was not attention, but age or other measures of physical status that were the best predictors.

To conclude, it seems that the type of game chosen to train visual attention is important, and ultimately a battery of games may be most effective - and fun! Skiing was the game that was best associated with visual attention, while Tai Chi was best associated with physical ability. Thus, not all games that appear to be associated with visual attention are strongly associated and other factors, such as physical ability, may predominate, e.g. for Stomp-it because of its association with age. It also seems that the associations between visual attention and gait and mobility, while present, are weak and often explained by age or other measures of physical function.

### Limitations

The study has some limitations and the results should be interpreted with caution. We used a cross-sectional design which means that association, not causation, can be implied. We do not know, for example, if poor attention affects a person’s gait, or whether in some way, poor gait changes attention. Only longitudinal studies can show which is the cause and which is the effect or whether there is a bi-directional effect. Additionally, the correlations in the models in this study, although significant, are not high, indicating that some factors that were not measured may influence the outcome variables and further cross-sectional or longitudinal studies are needed. Second, all data was collected by one researcher (MA). This may have lead to bias, as this individual was familiar with the hypotheses of the study. However, we do not think that this is likely, as data was collected in blocks (e.g. all the balance data at one time), and thus would be unlikely to remember the other measures from a particular participant in order to influence the data in any direction. Third, the mobility task was a possible limitation as it was a simple measure of time and stepping parameters along an unobstructed path. Using a more challenging mobility course with obstacles and light changes similar to changes that we experience in everyday life may have shown a better correlation with attention. Fourth, it was not possible to analyze the one-legged stance sway with the force plate as we had originally intended, as a large percentage of the participants could not maintain the 30 s stance resulting in insufficient data to analyze. So, the more basic measure of the total OLST time was used. This showed a ceiling effect as many participants could reach to the maximum standing time. Additionally, the video games were selected based on game availability, apparent visual requirements, and pilot testing which demonstrated that older adult participants could understand and successfully complete the game rather than on the basis of a theoretical model. Finally, the age range and the health status of our sample was more limited than in some studies. There were fewer older participants, and most were relatively healthy for their age which does not reflect the average of health status expected for this age group. So it is possible that they may not be totally representative of their whole age group. Their physical performance was similar or better than other studies that included healthy older participants [28].

## Conclusion

This study has investigated the relationships between visual attention, balance, mobility and Xbox® Kinect™ games performance. The results indicate that some visual attention measures are associated with high visual attention demand Xbox® Kinect™ games and can be a good predictor of the performance in this type of game. The study indicates that Xbox® or similar games may have potential for training visual attention as well as physical abilities, but the game chosen is critical. The evidence from this study also indicates that some balance and gait measures are associated with visual attention. This study enhances our understanding of visual attention and its association with other systems and could be a framework for further future studies and indicates that longitudinal studies may be useful to show the potential of these games to enhance mobility and balance in order to prevent falls in older adults.

## Data Availability

The datasets used and analysed during the current study are available from the corresponding author on reasonable request. Custom code was used for data processing; not available for distribution.

## Abbreviations

5MWT: Five-Meter Walking Test
AP: Anterior-posterior
CoP: Centre of Pressure
CPL: Cumulative Path Length
Max.: Maximum
ML: Medial-lateral
MoCA: Montreal Cognitive Assessment
MOT: Multiple Object Tracking
OLST: One-Legged Stance Test
QST: Bi-pedal Quiet Stance
SD: Standard Deviation
UFV: Useful Field of View
UFV-D: Useful Field of View-Dynamic
UFV-S: Useful Field of View-Static
VA: Visual Acuity
vel/leg: ratio of Velocity/Leg length
VIF: Variance Inflation Factor
